# Maternal betaine suppresses adrenal expression of cholesterol trafficking genes and decreases plasma corticosterone concentration in offspring pullets

**DOI:** 10.1186/s40104-019-0396-8

**Published:** 2019-11-19

**Authors:** Halima Abobaker, Yun Hu, Nagmeldin A. Omer, Zhen Hou, Abdulrahman A. Idriss, Ruqian Zhao

**Affiliations:** 1 MOE Joint International Research Laboratory of Animal Health & Food Safety, Nanjing Agricultural University, Nanjing, 210095 People’s Republic of China; 2 Key Laboratory of Animal Physiology & Biochemistry, Nanjing Agricultural University, Nanjing, 210095 People’s Republic of China; 3 College of Allied Medical Sciences, University of Nyala, 155 Nyala, Sudan

**Keywords:** Adrenal gland, Chicken, Cholesterol, Corticosterone, Maternal betaine, Steroidogenesis

## Abstract

**Background:**

Laying hens supplemented with betaine demonstrate activated adrenal steroidogenesis and deposit higher corticosterone (CORT) in the egg yolk. Here we further investigate the effect of maternal betaine on the plasma CORT concentration and adrenal expression of steroidogenic genes in offspring pullets.

**Results:**

Maternal betaine significantly reduced (*P* < 0.05) plasma CORT concentration and the adrenal expression of vimentin that is involved in trafficking cholesterol to the mitochondria for utilization in offspring pullets. Concurrently, voltage-dependent anion channel 1 and steroidogenic acute regulatory protein, the two mitochondrial proteins involved in cholesterol influx, were both down-regulated at mRNA and protein levels. However, enzymes responsible for steroid syntheses, such as cytochrome P450 family 11 subfamily A member 1 and cytochrome P450 family 21 subfamily A member 2, were significantly (*P* < 0.05) up-regulated at mRNA or protein levels in the adrenal gland of pullets derived from betaine-supplemented hens. Furthermore, expression of transcription factors, such as steroidogenic factor-1, sterol regulatory element-binding protein 1 and cAMP response element-binding protein, was significantly (*P* < 0.05) enhanced, together with their downstream target genes, such as 3-hydroxy-3-methyl-glutaryl-coenzyme A reductase, LDL receptor and sterol regulatory element-binding protein cleavage-activating protein. The promoter regions of most steroidogenic genes were significantly (*P* < 0.05) hypomethylated, although methyl transfer enzymes, such as AHCYL, GNMT1 and BHMT were up-regulated.

**Conclusions:**

These results indicate that the reduced plasma CORT in betaine-supplemented offspring pullets is linked to suppressed cholesterol trafficking into the mitochondria, despite the activation of cholesterol and corticosteroid synthetic genes associated with promoter hypomethylation.

## Background

Corticosterone (CORT) synthesis in the adrenal gland requires a continuous supply of cholesterol that is delivered to mitochondria as a substrate for steroidogenesis [1]. Mobilization of cholesterol from the cytoplasm into mitochondria is the rate-limiting step in steroidogenesis, which involves the actions of intermediate cytoskeleton filament proteins [2], such as vimentin (VIM) [3]. The role of steroidogenic acute regulatory protein (StAR) in shuttling cholesterol to the inner mitochondria membrane (IMM) has been well documented [4–8]. Also, the voltage-dependent anion channel (VDAC) not only regulates StAR activity at the outer mitochondrial membrane (OMM) [9, 10], but also interacts with StAR to assist the transfer of cholesterol into the IMM [11]. However, it is unknown whether feeding betaine to laying hens may affect the adrenal steroidogenesis in their progeny by targeting cholesterol shuttling machinery.

Steroidogenesis necessitates complex inter-communication among several subcellular compartments which demand regulation of cholesterol synthesis [12]. Sterol regulatory element-binding proteins (SREBP) drive the expression of genes involved in cholesterol synthesis [13]. In mammals, SREBP2 is more involved in cholesterol synthesis, while SREBP-1a is involved in both fatty acid and cholesterol biosynthesis [14]. In chickens, SREBP1 is highly homologous to SREBP1a in mammals [15]. However, less information is available regarding the role of SREBP1 in cholesterol synthesis in the adrenal glands of chickens.

Betaine is a feed additive to improve the growth performance in livestock [16] and to attenuate heat stress in poultry [17]. A number of studies have implicated a close relationship between betaine and glucocorticoids to coordinate the body functions for the recovery from stress. For instance, athletes subjected to intense exercise were detected increased betaine and decreased cortisol levels in the blood [18]. In another study, dietary betaine supplementation in sows during gestation reduced plasma cortisol level in neonatal piglets [19]. Also, dietary betaine alleviates heat stress in poultry by decreasing the plasma CORT level [20]. Nevertheless, the effect of betaine on plasma CORT level in the chicken is not consistent, which depends on the level and timing of betaine administration as well as the physiological status of the birds. Previously, we reported that dietary betaine supplementation to laying hens led to increased CORT deposition in the egg yolk [21]. Therefore, questions arise whether maternal betaine supplementation affects the adrenal CORT production and plasma CORT concentrations in chicken offspring later in life.

Betaine is an essential component of the methionine cycle [22] and can affect intracellular metabolism via modulating DNA methylation [23]. We reported previously that *in* *ovo* injection of betaine affects hepatic cholesterol metabolism in newly hatched chicks through epigenetic modifications [24]. However, it remains unknown whether maternal betaine would affect adrenal steroidogenic genes through epigenetic modifications in offspring chickens.

Therefore, here we sought to investigate the effect of maternal betaine supplementation on the adrenal expression of cholesterol trafficking genes in association with alterations in plasma corticosterone concentration; and to elucidate the mechanisms by determining the expression of methyl transfer genes and DNA methylation status on the promoter of relevant genes in offspring pullets.

## Methods

### Experimental design

One hundred and twenty Rugao yellow-feathered laying hens (1.4 ± 0.10 kg, mean ± SEM) were obtained from Poultry Institute, Chinese Academy of Agricultural Sciences, Yangzhou, China. Hens were randomly allocated into control (CON) and betaine-supplemented (BET) groups at 38 weeks of age, fed basal and betaine-supplemented diets, respectively, for 28 d. Betaine hydrochloride (98% purity, Skystone Feed Co., Ltd, Jiangsu, China) was added to the basal diet at the level of 0.5%. Laying hens were artificially inseminated at the 18^th^ and 24^th^ day post dietary treatment. Fertilized eggs were collected from 25^th^ to 28^th^ day post dietary treatment (200 eggs from each group) and incubated under standard conditions. Newly hatched chicks were raised following the standard established by the breeder. All the chicks were fed the same diet until 56 days of age when 15 pullets in each group were randomly selected and killed by rapid decapitation. The detailed procedure for rearing and slaughtering has been described in a previous publication [25]. Blood samples were taken from the jugular vein, and plasma was separated and stored at − 20 °C. Adrenal glands were dissected and rapidly frozen in liquid nitrogen and stored at − 80 °C for further analyses.

### Determination of plasma total cholesterol

Total cholesterol concentration in the plasma was determined with an automatic biochemical analyzer (Beckman coulter, AU2700), using a commercial kit purchased from Maccura Biology Co., Ltd. (ECH0103152, Chengdu, China). The intra-assay coefficient of variation was less than 3.0%.

### Plasma corticosterone assay

Corticosterone concentration in the plasma was measured with a commercial Enzyme Immunoassay (EIA) kit (No.ADI-900-097, Enzo, USA). The detection limit was 26.99 pg/mL and the intra-assay coefficient of variation was 8%.

### Total RNA isolation and real-time PCR

Total RNA was isolated from 20 mg adrenal samples using 1 mL of TRIzol reagent (Invitrogen, USA) and 2 μg of total RNA was subjected to RT-PCR as previously described [21]. The technical variations were normalized using beta-actin as an internal control. Samples were run in duplicates. Primers for real-time PCR (Table 1) were synthesized by Genewiz (Suzhou, China). Data were analyzed using the method of 2^−△△CT^ [26], and the results are presented as the fold change relative to the average value of the control group.

**Table 1:** Nucleotide sequences of specific primers used for real-time PCR

Target genes	GenBank accession	Primer sequences (5′ to 3′)	PCR products, bp
*StAR*	NM_204686.2	F:ATGGCATCCAAGGAGTGA	103
		R: GGGAGACAGAAGGGAACAG	
*CYP11A1*	NM_001001756.1	F: AGCACTTCAAGGGACTGAGC	147
		R:ACTTGGTCCCAACTTCCACC	
*3BHSD2*	XM_417988.5	F: CTGGAAGAAGATGAGGCGCT	249
		R: ACCTGTCACGTTGACTTCCC	
*CYP21A2*	>NM_001099358.1	F: CTTTGAGGCGTTCACGTTCC	169
		R: CTGGGACTCCACAAAGGCAT	
*CYP19A1*	NM_001364699.1	F: CATGCACCCAATAGAAAGGCA	130
		R: GCATTTCTTAAAGTGACTGCAAAC	
*CYP17A1*	NM_001001901.2	F: TCTGCTCCCTCTGCTTCAA	250
		R: AGGTCCCTCACAGTGTCCC	
*SF-1*	XM_015279334.1	F: TCTTCCTGAATTTCCCTT	151
		R: TGAACATCCCATCTAGTGA	
*CREB*	XM_015294628.2	F: GTCAGACACACCAGAGCCTT	128
		R: CATTCCTGCTCCCCTTCCTC	
*BHMT*	XM_414685.3	F: CGAGTGGGACGGCTTCTT	144
		R: AGGCGATAGGTGTCAGGGA	
*AHCYL1*	NM_001030913.1	F:TGGTGTTGTTGGGGGAGAT	227
		R:CCCCATCAATACTCATCCAAC	
*DNMT1*	NM_206952.1	F:TGATAYGTTGGATGAGTATTGATGG	264
		R: AAAAAAACTCTCACTCAACTCCAC	
*GNMT1*	XM_015283546.1	F: GGAGGAGGGCTTCCAAGTGA	140
		R: GCTCCAGCGTCAGCCAGTT	
*VIM*	AH002482.2	F: CTTTGCCCAGTGCTGTAGTC	171
		R: AAACACGGGCTGTCAGTCA	
*VDAC1*	NM_001033869.2	F: GAACAGGGACGGGGACAG	136
		R: CAGACTTGCCCAGATCAGCA	

### Total protein extraction and Western blot analysis

Total protein was extracted from 15 mg frozen adrenal samples, as previously described [24]. BCA Protein Assay kit (No. 23225, Thermo Scientific) was used to measure protein concentrations following manufacturer’s instructions. Fifty micrograms of protein were used for electrophoresis on a 10% SDS-PAGE gel. Western blot analysis was performed following the protocols provided by the manufacturers. Tublin-β (AP0064, Bioworld, USA, diluted 1:5,000) was used as an internal reference. VersaDoc 4000MP system (Bio-Rad, USA) was employed to capture the images and Quantity One software (Bio-Rad, USA) was used to analyze the band density.

### Methylated DNA immunoprecipitation (MeDIP) analysis

MeDIP analysis was executed according to a previous publication [27]. A small aliquot of MeDIP DNA and control input DNA was used to amplify the proximal promoter sequence of chicken *VIM*, *VDAC1*, *StAR*, *CYP11A1*, *CYP21A2*, *SF-1*, *CREB* and *SREBP1* genes by RT-PCR with specific primers listed in Table 2.

**Table 2:** Nucleotide sequences of primers used for MeDIP

Target genes	Primer sequences (5′ to 3′)	PCR products, bp
*StAR*		
Segment 1	F: CAGGGACACCTCGGTTCTTC	177
	R: GCTGTTATCCCAATGGAGCG	
Segment 2	F: CTCGGGGTCTTTCATTGCCA	129
	R: CAGGGAGCAGGCGATAAGAT	
Segment3	F: GGCGGTTTCTGTTCAGAGGT	160
	R:: CAGAGCAACACCCCAAACAC	
Segment 4	F: GGCGGTTTCTGTTCAGAGGT	196
	RACAGAGCAACACCCCAAACAC	
*CYP11A1*		
Segment 1	F: TAAGGGCCGTGTTTTGGAGG	194
	R: TGGGGACTCAGCAGATTTCG	
Segment 2	F: AACTGACAGCGTAATGCCCA	164
	R: AAAGAGGGGGTTGGAAACGG	
Segment3	F: CAGGGTATGGGTTGCAGGTT	116
	R: GTGGAAAACCCCCATCGTCT	
*CYP21A2*	F: CTCAACGTGAGATCTGGGGG	148
	R: TTGAAAGGTCCAGTTGGGGG	
*VIM*		
Segment 1	F: GTGGGGACGCCGCTCTT	181
	R: GGGTGCTGGACGTGATGTAG	
Segment2	F: GGGACGCCGCTCTTCTT	110
	R: GAGGAGTTCTTGCTGCTGGT	
*SF-1*		
Segment 1	F: CATTTACCCCCGCAAACACC	187
	F: CTCTAGCAGGTTCAAGGTCCC	
Segment 2	F: TATCGCCAAAGTCCTCACCG	193
	R: CTCCATCCACGCGGCTTATC	
*VDAC1*	F: AAGTGATCTGCCTGTCTCGG	144
	R: TTGAGGCTGGGAGCAAATGT	

### Statistical analysis

Data are presented as means ± SEM. The normality and homogeneity of variances were checked before using parametric analyses. When the data were not normally distributed, the log_10_ transformation was performed before statistical analysis. T-test for independent samples was performed for comparisons using SPSS 20.0 for windows. The differences were considered statistically significant when *P* < 0.05.

## Results

### Plasma corticosterone, total cholesterol and adrenal expression of cholesterol trafficking genes

**Fig. 1:**
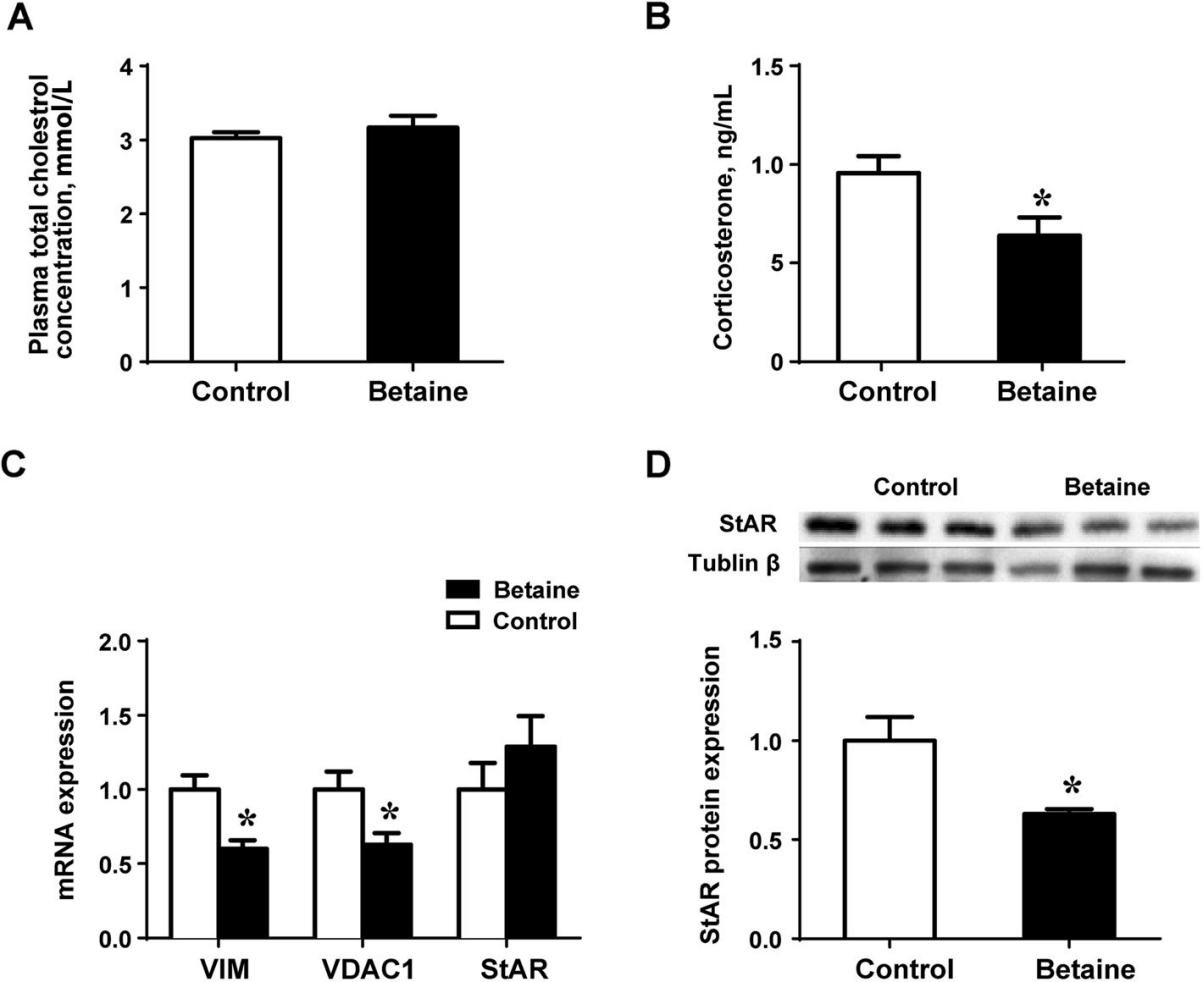
Plasma corticosterone, total cholesterol and adrenal expression of cholesterol trafficking genes in pullets. **a**) Plasma total cholesterol; **b**) Plasma corticosterone **c**) mRNA abundance of genes involved in cholesterol trafficking **d**) StAR protein expression. Values are means ± SEM, *P < 0.05, compared with control for the mRNA (*n* = 7), for protein expression (*n* = 4)

Maternal betaine did not affect plasma total cholesterol in the young pullets (Fig. 1a). However, plasma corticosterone concentration was significantly decreased (*P* < 0.05) in the betaine group, together with the mRNA abundance of *VIM* and *VADC1* genes (Fig. 1b and c). StAR protein content was significantly (*P* < 0.05) down-regulated (Fig. 1d) in the adrenal glands of pullets derived from betaine-supplemented hens, although no difference was detected at the mRNA level.

### Adrenal expression of cholesterol metabolic genes

**Fig. 2:**
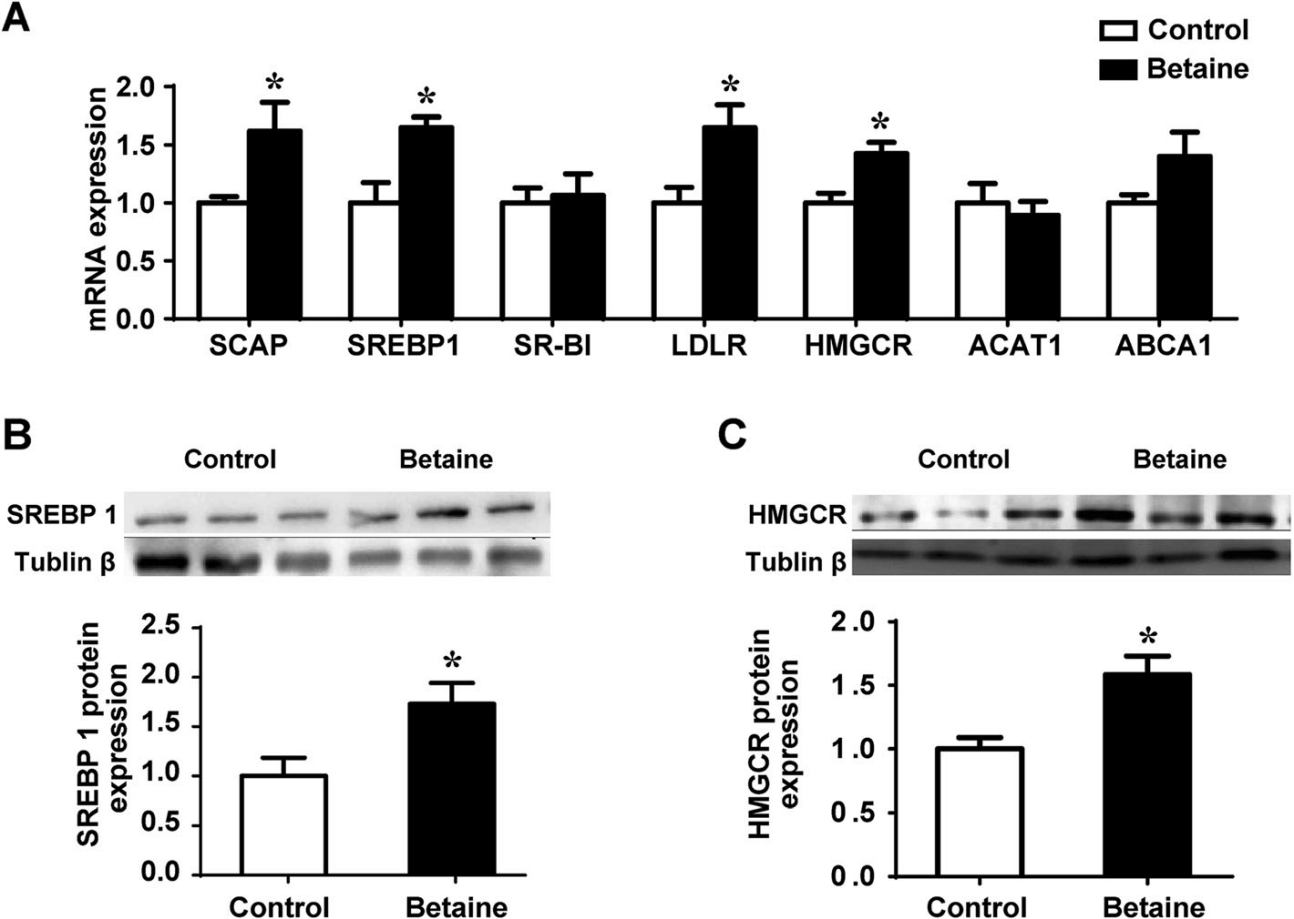
Adrenal expression of cholesterol metabolic genes in pullets. **a**) Cholesterol biosynthetic genes mRNA expression; **b**) SREBP1 protein expression; **c**) HMGCR protein expression. Values are means ± SEM, ^*^P < 0.05, compared with control for the mRNA (*n* = 7), for protein expression (*n* = 4)

*SCAP*, the escort protein that engages SREBP in *de novo* cholesterol synthesis, and low-density lipoprotein receptor (LDLR) that involved in cholesterol uptake, were both significantly (*P* < 0.05) up-regulated at mRNA level (Fig. 2a). Also, both SREBP1 and 3-hydroxy-3-methyl-glutaryl-coenzyme A reductase (HMGCR), the rate-limiting enzyme for cholesterol synthesis, were significantly (*P* < 0.05) increased at both mRNA (Fig. 2b) and protein levels (Fig. 2c) in the adrenal glands of pullets derived from betaine-supplemented hens.

### Adrenal expression of transcription factors and downstream corticosteroid biosynthetic enzymes

**Fig. 3:**
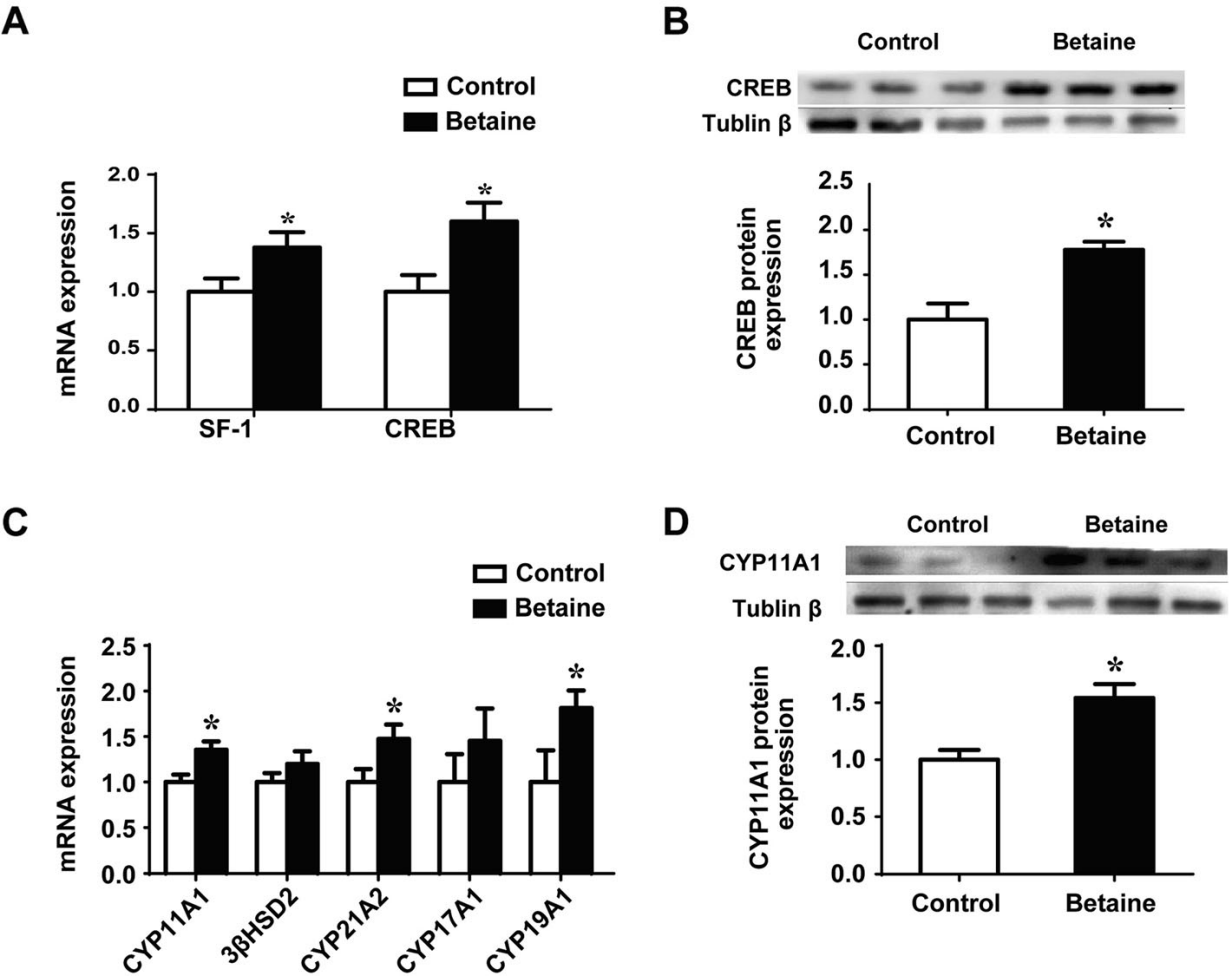
Adrenal expression of transcription factors and downstream corticosteroid biosynthetic enzymes in pullets. **a**) *SF-1* and *CREB* mRNA expression **b**) CREB protein expression; **c**) mRNA expression of corticosteroid biosynthesis enzymes; **d**) CYP11A1 protein expression. Values are means ± SEM, *P < 0.05, compared with control for the mRNA (*n* = 7), for protein expression (*n* = 4)

The adrenal expression of *SF1* was significantly (*P* < 0.05) up-regulated at mRNA level, while that of CREB was significantly (*P* < 0.05) increased at both mRNA and protein levels (Fig. 3a and b) in pullets derived from betaine-supplemented hens. Additionally, both mRNA abundance (Fig. 3c) and protein content (Fig. 3d) of CYP11A1 were significantly (*P* < 0.05) increased in the betaine group. Concurrently, *CYP21A2* and *CYP19A1* mRNA abundances were also significantly elevated (*P* < 0.05), in comparison with their control counterparts (Fig. 3c).

### Adrenal expression of methionine metabolic genes

**Fig. 4:**
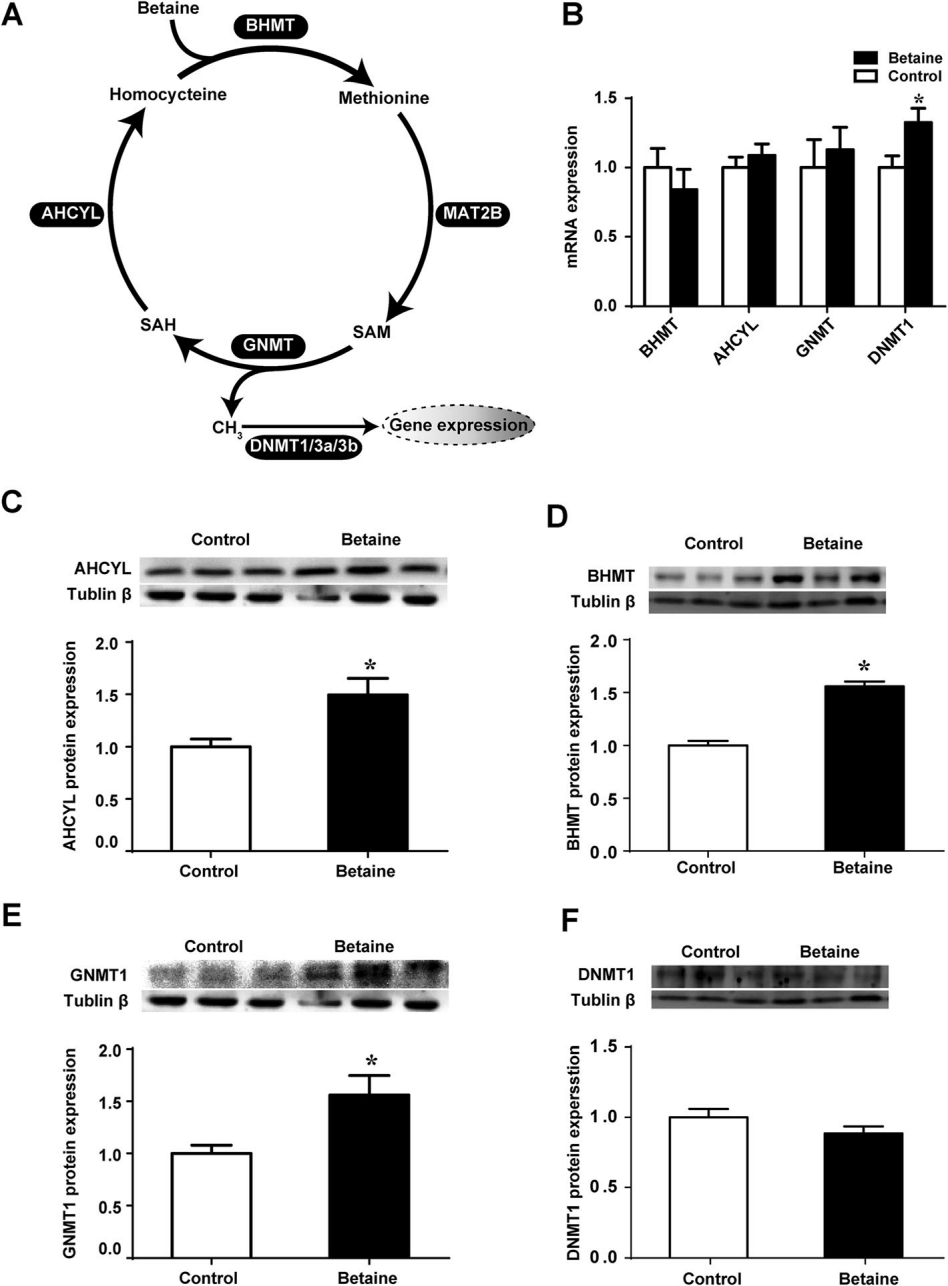
Adrenal expression of methionine metabolic genes in pullets. **a**) Schematic diagram of methionine cycle; **b**) Adrenal expression of key enzymes involved in methionine metabolic cycle at the mRNA; **c**) AHCYL protein expression; **d**) BHMT protein expression; **e**) GNMT1 protein expression; **f**) DNMT1 protein expression. Values are means ± SEM, *P < 0.05, compared with control. mRNA (*n* = 7), for protein expression (*n* = 4)

Enzymes involved in the methionine cycle are shown in the schematic diagram (Fig. 4a). No changes were detected for *AHCYL*, *BHMT* or *GNMT* at the level of mRNA (Fig. 4b), but all three of them were significantly up-regulated (*P* < 0.05) at the protein levels (Fig. 4c, d and e). Interestingly, *DNMT1* mRNA was significantly higher (*P* < 0.05) in the betaine group compared with the control group, but no alterations were detected at the protein level (Fig. 4f).

### DNA methylation of cholesterol trafficking gene promoters

**Fig. 5:**
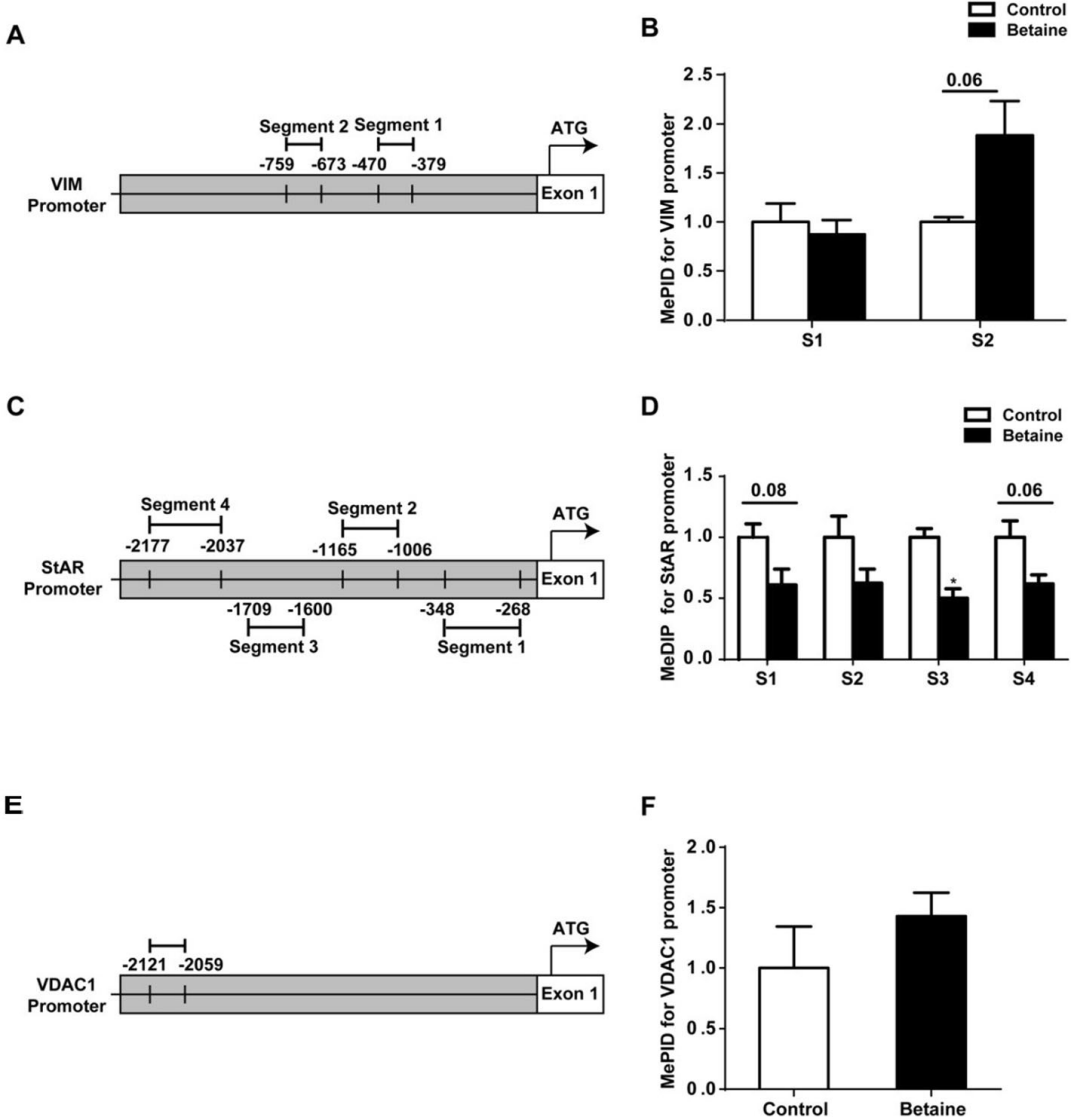
DNA methylation of cholesterol trafficking gene promoters in adrenal glands of pullets. **a**) Schematic diagram showing the amplified segments (S) on the promoter sequence of *VIM*. **b**) DNA methylation status on the promoter of *VIM*; **c**) Schematic diagram showing the amplified *StAR* segments (S); **d**) DNA methylation status on the promoter of *StAR*; **e**) Schematic diagram showing the amplified *VDAC1* (S); **f**) DNA methylation status on the promoter of *VDAC1*. Values are means ± SEM, *P < 0.05, compared with control (*n* = 3)

Different segments (S) of the promoter sequences of chicken *VIM* (Fig. 5a)*, StAR* (Fig. 5c)*,* and *VDAC1* (Fig. 5e) were analyzed with MeDIP-PCR technique. Maternal betaine tended (*P* = 0.06) to increase DNA methylation level on the S2 of *VIM* gene promoter (Fig. 5b), while S3 on *StAR* gene promoter was significantly (*P* < 0.05) hypomethylated in betaine group, together with S1 (*P* = 0.08) and S4 (*P* = 0.06) that showed a tendency of hypomethylation (Fig. 5c). No alteration was detected for the methylation status on *VDAC1* gene (Fig. 5f).

### DNA methylation of transcription factors and steroidogenic genes promoters

**Fig. 6:**
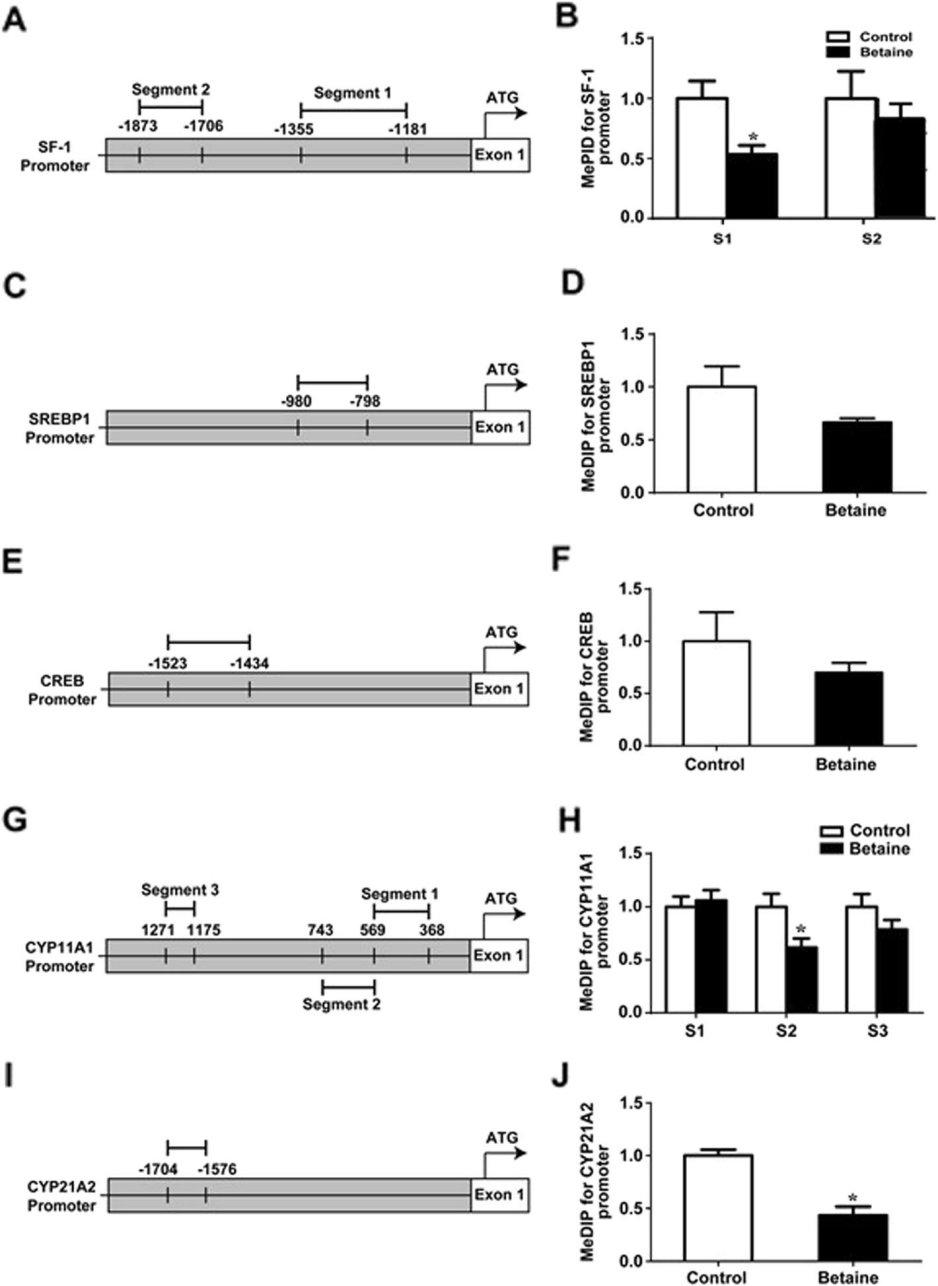
DNA methylation of transcript factors and steroidogenic genes promoters in adrenal glands of pullets. **a**) Schematic diagram showing the amplified segments (S) on the promoter sequence of *SF-1*. **b**) DNA methylation status on the promoter of *SF-1*; **c**) Schematic diagram showing the amplified *CREB*; **d)** DNA methylation status on the promoter of *CREB*. **e**) Schematic diagram showing the amplified segments (S) on the promoter sequence of *SREBP1*. **f**) DNA methylation status on the promoter of *SREBP1*; **g**) Schematic diagram showing the amplified *CYP11A1*; **h**) DNA methylation status on the promoter of *CYP11A1*.; **i**) Schematic diagram showing the amplified *CYPA2*(S); **j**) DNA methylation status on the promoter of *CYP21A2*. Values are means ± SEM, *P < 0.05, compared with control (*n* = 3)

Moreover, the promoter sequences of genes coding for relevant transcription factors, including *SF-1* (Fig. 6a)*, SREBP1* (Fig. 6c) and *CREB* (Fig. 6e), as well as steroidogenic enzymes, such as *CYP11A1* (Fig. 6g) and *CYP21A2* (Fig. 6i), were also analyzed. S1 of the SF-1 gene promoter showed a significant (*P < 0.05*) hypomethylation in betaine group (Fig. 6b), while no differences were detected for either *SREBP1* or *CREB* gene promoters (Fig. 6d and f). S2 of the *CYP11A1* promoter (Fig. 6h), together with *CYP21A2* gene promoter sequence (Fig. 6j), was significantly (*P* < 0.05) hypomethylated in the adrenal gland of pullets derived from betaine-exposed hens.

## Discussion

Developmental origins of health and disease (DOHaD) has attracted attention in the field of biomedical research. Several studies demonstrated the effect of maternal nutrition during pregnancy on serum CORT level in rat offspring. For example, feeding dams with a protein-restricted diet led to increased plasma CORT level in rat offspring [28]. Also, maternal high-fructose consumption increases plasma CORT concentration in adult rat offspring [29]. The adverse effects of maternal malnutrition on offspring are considered to be mediated, at least partly, by dysregulated CORT production.

In this study, maternal betaine reduced plasma CORT concentration in offspring chicken, which was associated with suppressed expression of cholesterol trafficking genes, such as *VIM,* *StAR* and *VDAC1,* in adrenal glands of the pullets. Shen et al. [30] found a marked reduction of plasma CORT and progesterone levels in *VIM* null mice. Likewise, shuttling cholesterol into the mitochondria requires the interplay of VDAC with StAR [19]. The abundant presence of VDAC on cholesterol ester (CE)-enriched LDs points to the role of VDAC in promoting CE mobilization into the mitochondria [31]. Also, CORT production was depressed in *StAR* null mice [32]. In this study, no alteration was detected in plasma total cholesterol concentration, and the adrenal content of cholesterol was not measured due to limited sample size. However, the consistent suppression of all the three cholesterol trafficking genes led to an assumption that maternal betaine decreased the plasma CORT concentration in offspring pullets via inhibiting the process of cholesterol shuttling to mitochondria for CORT biosynthesis.

SREBP1 serves as a cholesterol sensor to activate or suppress the cholesterol biosynthesis to maintain the intracellular cholesterol homeostasis [33]. Shortage of intracellular cholesterol will activate the cholesterol synthesis loop by LDLR and HMGCR. Conversely, when intracellular cholesterol concentrations are high, this process will be inhibited [34]. In this study, the SREBP1-mediated cholesterol feedback machinery was activated in the adrenal gland of pullets exposed prenatally to betaine. Both SCAP and SREBP1, alongside its downstream HMGCR and LDLR, were up-regulated. SREBPs triggering requires SCAP and many transcription factors, such as CREB and SF-1 [10, 35]. In this study, maternal betaine also significantly up-regulated *SF1* at the mRNA level and CREB at both mRNA and protein levels. In accordance with these findings, the downstream enzymes responsible for steroidogeneses such as cytochrome P450 family members, including *CYP11A1*, *CYP21A2* and *CYP19A1*, were all significantly up-regulated in the betaine group. Together, our results indicate that the increase of SREBP1-mediated cholesterol biosynthesis and SF-1-activated steroidogenesis in the adrenal gland of young pullets from the betaine group may represent a compensatory adaptation to the decrease in plasma CORT concentration.

DNA methylation is recognized as a fundamental mechanism in the regulation of steroidogenic genes [36]. For instance, prenatal caffeine exposure decreased *SF-1* mRNA expression in association with DNA hypermethylation in the *SF1* gene promoter [37]. DNMTs are responsible for DNA methylation required for normal cellular metabolism [38]. In this study, maternal betaine enhanced the expression of the methionine cycle and methyl transfer enzymes such as GNMT1, AHCYL1 and DNMT1. Up-regulation of *SF1, CYP11A1 and CYP21A2* genes coincided with hypomethylation of their promoters. However, a clear link between mRNA expression and promoter DNA methylation was missing for cholesterol trafficking genes. Further investigations are required to unravel the mechanisms by which maternal betaine suppresses the expression of cholesterol trafficking genes in adrenal glands of offspring pullets.

## Conclusions

This study provides evidence that maternal betaine decreases plasma CORT level with suppressed expression of cholesterol trafficking genes in the adrenal gland of offspring chickens. The lower basal plasma CORT concentration may allow pullets better coping with stress during the laying period. However, future studies are required to evaluate the long-term effect of maternal betaine on the laying performance of their female progeny.

## Data Availability

The datasets used and analysed during the current study available from the corresponding author upon request.

## Abbreviations

ACTH: Adrenocorticotrophic hormone
AHCYL1: Adenosylhomocysteinase-like 1
BHMT: Betaine-homocysteine methyltransferase
CREB: CAMP response element-binding protein
CYP: Cytochrome P450
DNMTS: DNA methyltransferases
GNMT: Glycine N-methyltransferase
HPA: Hypothalamic-pituitary-adrenal
HSD: Hydroxysteroid dehydrogenases
IMM: Inner mitochondrial membrane
MeDIP: Methylated DNA immunoprecipitation
OMM: Outer mitochondrial membrane
SAM: S-adenosylmethionine
SF-1: Steroidogenic factor-1
SREBPS: Sterol regulatory element-binding protein
StAR: Steroidogenic acute regulatory protein
VDAC1: Voltage-dependent anion channel 1
VIM: Vimentin
